# Diagnosis and treatment of 2 cases with cryptogenic stroke due to patent foramen ovale in children: A case report

**DOI:** 10.1097/MD.0000000000039986

**Published:** 2024-10-04

**Authors:** Jie Gong, Huayong Zhang, Changjian Li, Yong Zhang

**Affiliations:** a School of Medicine, Jianghan University, Wuhan, China; b Department of Cardiology, Wuhan Children’s Hospital (Wuhan Maternal and Child Healthcare Hospital), Tongji Medical College, Huazhong University of Science and Technology, Wuhan, China

**Keywords:** arterial ischemic stroke, children, cryptogenic stroke, interventional operation, patent foramen ovale

## Abstract

**Rationale:**

Arterial ischemic stroke is a general term for necrosis of brain tissue due to insufficient blood supply to the brain from various causes. About 30% of these cases are of unknown origin and are known as cryptogenic strokes (CS).

**Patient concerns:**

We report 2 female patients, one 5 years and 5 months old and the other 6 years old. Both children had clinical manifestations of CS, and after ruling out other possible etiologies, we finally suspected that CS was associated with patent foramen ovale (PFO).

**Diagnoses:**

Case 1 was diagnosed with PFO, paradoxical embolism, and third-degree atrioventricular block, and case 2 was diagnosed with PFO, paradoxical embolism, and refractory mycoplasma pneumonia.

**Interventions:**

Case 1 underwent permanent pacemaker placement at the same time as PFO closure. Case 2 underwent conservative anticoagulation with poor therapeutic results and subsequently underwent PFO closure.

**Outcomes:**

Patient 1 underwent PFO closure, which resulted in relief of neurologic symptoms and no recurrence of neurologic symptoms after 10 months of follow-up. In case 2, the child’s neurologic symptoms improved after PFO closure.

**Lessons:**

Although most children with PFO do not require targeted interventions, a few cases involving PFO and CS may benefit from closure of the foramen ovale.

## 1. Introduction

Patent foramen ovale (PFO) is a normal atrial blood flow pathway during fetal life. It is also a necessary passageway for the fetus to survive. The PFO will anatomically close 2 months after birth and reach anatomically complete closure within 1 year.^[1]^ However, PFOs also remain in about 25% to 30% of adults, and when right heart chamber pressures increase (e.g., Valsalva maneuver), PFOs can open, causing right-to-left shunts in the atria.^[2]^ Arterial ischemic stroke (AIS) is a general term for necrosis of brain tissue due to insufficient blood supply to the brain from a variety of causes. About 30% of AIS cases have an unknown cause, called cryptogenic stroke (CS). Some patients with CS have coexisting PFOs, and it is currently believed that there is a correlation between CS and PFOs.^[3,4]^ The mechanism by which a PFO triggers CS has been controversial, and 3 potential mechanisms by which a PFO triggers CS in stroke are currently thought to be possible. Three possible mechanisms include paradoxical embolization (PE), “insitu” thrombosis, and arrhythmias.^[5]^ PE is the most common cause of CS. PE is when a PFO establishes a direct connection between right and left heart blood flow, and emboli from the veins or right atrium may enter the arterial circulation directly and reach the cerebral arteries to obstruct blood flow, leading to AIS. In addition to cerebral embolism, it can lead to myocardial infarction, gastrointestinal ischemia, renal infarction, and peripheral arterial embolism.^[6]^ The in situ thrombus hypothesis refers to the formation of a thrombus in the atrial septum, the mechanism of which may be related to the stagnation of blood flow in the atria and the pressure difference between the atria.^[7]^ Another hypothesis suggests that PFO may cause abnormal left atrial electrical activity, which in turn causes arrhythmias such as premature atrial contractions. Abnormal left atrial electrical activity in a child can lead to thrombosis, which can cause CS.^[8]^

Although the exact mechanism by which PFO leads to CS is currently unknown, many studies have confirmed the superiority of transcatheter occlusion with dual-disc design devices over medication alone in preventing stroke recurrence in selected patients.^[9–12]^ Guidelines have recommended the choice of treatment modality for patients > 18 years of age.^[13]^ As pediatrics has evolved, few children with CS combined with PFOs have required decision-making. However, the mechanism and etiology of thrombosis in children are not the same as in adults,^[14]^ and direct reference to adult guidelines is somewhat inadequate. There are fewer reports of PFO combined with CS in children, and we report 2 cases of PFO with paradoxical embolism and ultimately successful occlusion in our center to provide some reference for pediatric colleagues.

## 2. Case presentation

**Figure 1.**
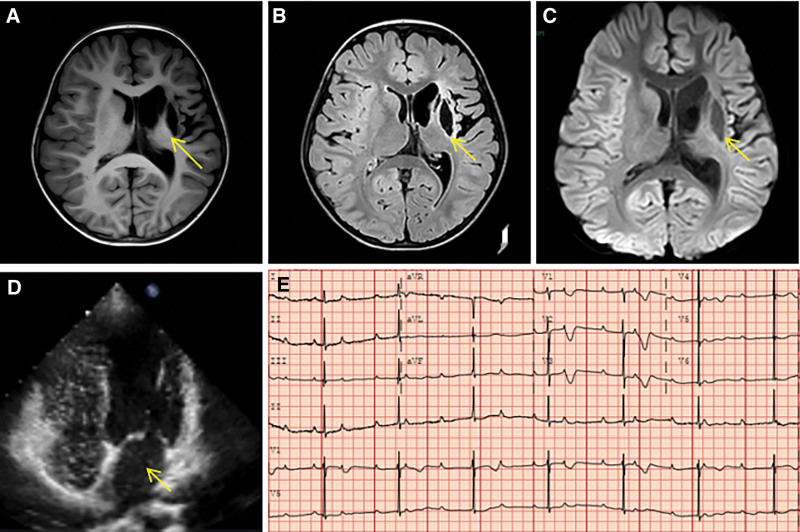
Examination results of case 1. (A) Cranial magnetic resonance imaging: T1 sequence: infarction site was uniformly low signal equal to the cerebrospinal fluid signal (yellow arrow); (B) Cranial T2 flair sequence: the lesion was low signal in the medial part of the infarction site, and high signal in the lateral part of the infarction site (yellow arrow), which was a typical old infarction lesion; (C) Diffusion-weighted imaging sequence: low-signal shadow at the lesion site; (D) Right heart acoustic imaging: equal amount of medium-volume microbubbles in the left heart (yellow arrows); (E) Electrocardiogram: third-degree atrioventricular block.

Case 1, 6 years 5 months, female. She was admitted to the hospital on June 28, 2023, for “the left side of the mouth distorted, right side of the limbs hemiplegia for 3 years, progressive aggravation for 2 days.” This child was found to have third-degree atrioventricular block (III AVB) at birth but did not seek regular medical attention. On March 29, 2020, the child presented with the left side of the mouth and the right side of the limb hemiplegia without any apparent cause. From March 4, 2020, to October 4, 2020, she was hospitalized in another hospital with the diagnosis of “left-sided cerebral infarction and III AVB.” She was treated with “low molecular heparin anticoagulation therapy.” She was discharged from the hospital on chronic aspirin to fight platelet clumping. Three days before admission, the corners of her mouth were tilted, and her right limb dyskinesia was worse than before. So, she went to Wuhan Children’s Hospital. Physical examination after admission: Heart rate 48 beats per minute, normal heart sounds, arrhythmia, impaired mobility of the right limb, inability to rotate the right hand anteriorly and posteriorly, poor active grasping, poor flexion of the right elbow, markedly increased muscle tone of the right elbow and the right ankle, markedly abnormal postures, inability to walk alone. The Cranial magnetic resonance imaging shows an abnormal signal in the left frontotemporal-parietal lobe, insula, basal ganglia, and centrum semiovale (Fig. 1A–C). Electrocardiogram (Fig. 1E) and ambulatory electrocardiogram: III AVB, mean heart rate 43 beats per minute. Carotid ultrasound showed no significant abnormalities. Laboratory tests, including blood tests, liver function, kidney function, cardiac enzymes, electrolytes, coagulation function, thyroid function, troponin, erythrocyte fragility test, immunization set, and whole blood hemoglobin assay, showed no significant abnormalities.

This child was admitted to the hospital after ruling out myocarditis, intracranial vasculopathy, extracranial vasculopathy (e.g., carotid artery stenosis), and hemoglobinopathies, among other possible known causes of cerebral emboli. The child had a history of unexplained stroke, difficulties moving their limbs, and recurring paradoxical embolism. She was diagnosed with a III AVB, with a mean heart rate of <50 beats per minute and a positive result on a right heart acoustic angiogram (class II) (Fig. 1D). She was deemed to require a permanent pacemaker and surgical closure of the PFO. She underwent a single-chamber permanent pacemaker placement and PFO blockage on July 1, 2023, after completing the preoperative investigations, and there were no contraindications to the procedure. She continued to take oral aspirin (3–5 mg/kg/d) to fight platelet coagulation for 6 months after the surgery was completed. She was followed up for 10 months without another stroke.

**Figure 2.**
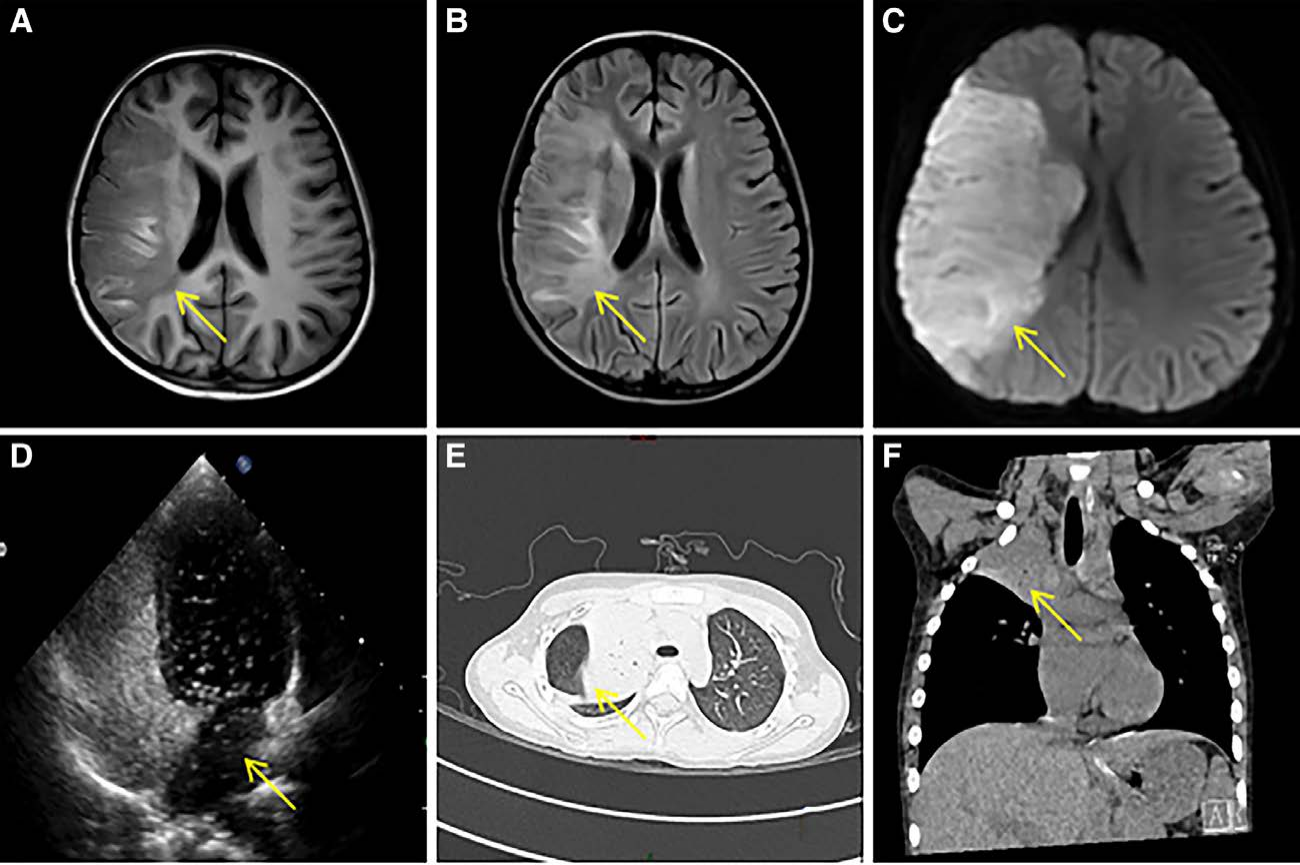
Examination results of case 2. (A) Cranial MRI: T1 sequence, ischemic stroke is a low-signal shadow; (B) Cranial MRI: T2 Flair sequence, ischemic stroke is a white high-signal shadow (yellow arrows); (C) Cranial MRI: Diffusion-weighted imaging sequence, ischemic stroke is a white high-signal shadow (yellow arrows), which is a typical manifestation of acute ischemic stroke; (D) The acoustic image of the right heart shows a large number of microbubbles in the left heart (yellow arrows); (E, F) The computed tomography of lungs: a large solid image of the right lung can be seen (yellow arrows). MRI = magnetic resonance imaging.

Case 2: A 6-year-old female was admitted to the hospital on August 18, 2022, with a persistent fever and cough for 3 days. The patient’s symptoms were recurrent high fever with an irritating dry cough. Physical examination revealed pharyngeal congestion, decreased breath sounds in the right lung, and no rales in both lungs. The chest computed tomography indicates pneumonia and solid changes in the upper lobe of the right lung; the distal bronchus of the upper lobe of the right lung is poorly visible. Pathogenetic testing suggested mycoplasma IgM positivity. Therefore, we diagnosed her with “mycoplasma pneumonia” and treated her with intravenous cefmetazole and oral azithromycin to combat the infection. Two days later, the child experienced a sudden onset of headache, weakness on the left side of the body, and a crooked mouth. The child was then transferred to the Pediatric Intensive Care Unit. During the physical examination, the following was noted: crooked mouth, soft neck, cheerful Bartholomew sign on the left side, negative Bartholomew sign on the right side, absence of knee tendon reflexes bilaterally, muscle strength of the left limb classified as level I to II (Lovett muscle grading standard), muscle strength of the right limb classified as level IV to V, slightly reduced muscle tone in the left limb, and normal muscle tone in the right limb. The laboratory tests showed white blood cell counts 10.66 (10^9^/L), neutrophil percentage 86.1 (%), C-reactive protein 98 (mg/L), erythrocyte sedimentation rate 21 (mm/h), creatine kinase-MB 37 (U/L), lactate dehydrogenase 922 (U/L), D-dimer 8.29 (mg/L), ferritin 628.19 (ng/ml), and procalcitonin 1.420 (ng/mL). The electrolytes, liver function, quantitative whole blood hemoglobin, blood lipids, immunization set, carotid ultrasound, and electrocardiogram showed no significant abnormalities. Repeat computed tomography of the lungs revealed bilateral pneumonitis of the lungs with partial solidity (Fig. 2E and F), perfect acoustic contrast of the right heart: positive/class III (Fig. 2D), a large number of microbubbles visible in the left heart both in a calm state and after forceful breath-holding (>30 microbubbles/frame visible in the left heart cavity). A cranial magnetic resonance imaging revealed acute cerebral infarction in the right frontotemporal-parietal lobe and right basal ganglia region, with occlusion of the right middle cerebral artery (Fig. 2 A–C). The child then received intravenous gammaglobulin, low molecular heparin anticoagulation, bronchoscopic alveolar lavage, and other therapeutic measures. After her temperature stabilized, the child was discharged from the hospital (September 15, 2023) and has been taking oral aspirin for over 2 months since discharge.

On November 25, 2023, the child was seen again with continued weakness of the left limb and a crooked mouth. In conjunction with the child’s medical history and right heart acoustic angiographic findings, focal cerebral arterial lesions, vascular malformations, vasculitis, structural cardiac lesions, myocarditis, endocarditis, and hematologic origin were ruled out. A final consideration is hypercoagulability due to infection, venous thrombosis, and venous thrombus entering the left cardiac system through the foramen ovale, leading to cerebral embolism. This child underwent percutaneous PFO sequestration on November 27, 2023, at the Department of Cardiovascular Medicine, Wuhan Children’s Hospital. After interventional occlusion, the child’s neurological symptoms improved, and no recurrence of stroke was seen at the later outpatient clinics at 3 months, 6 months, and 1 year.

## 3. Discussion

The child in case 1 of this study presented with the left side of the mouth and right limb hemiplegia for 3 years with progressive exacerbation for 2 days. This child has a history of III AVB with a history of ischemic stroke and is considered to have a recurrent ischemic stroke. She had a positive foaming test and underwent pacemaker placement and closure of the foramen ovale after ruling out other potential triggers. In case 2, the child presenting with ischemic stroke due to a severe pneumonia infection also had a positive foaming test. We ruled out potential causative factors and considered ischemic stroke due to PE. We completed a preoperative evaluation with full parental consent, and there were no contraindications to surgery in this child, who subsequently underwent percutaneous transluminal occlusion. The last 2 children recovered well after percutaneous perforation foramen ovale occlusion blockage. The child had III AVB combined with paradoxical embolism, suggesting the possibility of paradoxical embolism in III AVB combined with PFO. The child had III AVB combined with paradoxical embolism, suggesting the possibility of paradoxical embolism in III AVB combined with PFO. Simantirakis et al^[15]^ showed that based on the fact that bradycardia combined with atrioventricular separation may lead to increased circulatory time and blood flow stagnation, predisposing to venous thrombosis, thus raising the hypothesis of whether or not III AVB may also lead to pulmonary embolism, which has not yet been tested in clinical or experimental studies. We hypothesized that III AVB results in stagnant blood flow due to prolonged bradycardia. Some children are at risk of venous thrombosis due to the presence of cardiac disease and low levels of plain exercise, and thus, the risk of venous thrombosis. When a PFO is present, emboli from the venous system may enter the arterial system through the PFO to form a cerebral embolism. Of course, this part of the case is underreported and has some bias. We observed only a correlation between cerebral infarction and PFO, not a causal relationship. There are many causes of cerebral embolisms. Extreme cases such as bradycardia leading to dislodgment of emboli in the cardiac chambers cannot yet be excluded entirely. However, the highlights of our case are, firstly, it is worth exploring whether children with degree III AVB need to be managed aggressively and whether they should be concerned about the presence of coaptation of the foramen ovale in addition to the reference to heart rate.^[16]^ Second, unlike adults, pediatric strokes require vigilance for congenital disorders such as AVB and PFO. Aggressive evaluation of ambulatory electrocardiograms, cardiac ultrasound, and even foam labs are mandatory for children with pediatric strokes.

In the second case, the child suffered a cerebral infarction during treatment for mycoplasma pneumonia, and the family reported that the child had been in good health. We hypothesized whether mycoplasma pneumonia led to thrombosis, paradoxical embolism, and cerebral infarction through the PFO. Previous studies have found severe mycoplasma infections in children can lead to hypercoagulable states.^[17]^ Among them, Chaturvedi^[18]^ showed that coagulation abnormalities may promote the development of ectopic embolism in patients with PFO combined with CS and that children with Mycoplasma pneumonia combined with vascular embolism often present with elevated D-dimer in early life. Increased D-dimer levels reflect hypercoagulability, intravascular thrombosis, and secondary hyperfibrinolysis and have been widely used in diagnosing thrombotic diseases, assessing efficacy, and prognosis. It has been found that D-dimer is higher than 5 mg/L in all pediatric patients with pulmonary embolism.^[19]^ The elevation of D-dimer can lead to embolism in the venous system, and thus, emboli enter the arterial system through the PFO to form cerebral embolism. In our case, the child was admitted to the hospital with a D-dimer of 8.29 mg/L, which was already significantly elevated. This suggests the possibility of a pulmonary embolism or a hypercoagulable state of the blood. Cerebral infarction winds up being significantly increased when there is a paradoxical embolism associated with a PFO. Of course, D-dimer is not a specific indicator. In our clinic, we found that some children with mycoplasma infection were combined with pulmonary embolism and PFO. However, paradoxical embolism did not occur, which suggests that more studies are needed to confirm whether cerebral embolism occurs in mycoplasma pneumonia and PFO has a causal relationship. Nevertheless, the purpose of our report is that although thrombosis treatment in children is based on adult data, the fact is that venous thrombosis in children differs from that in adults in that thrombosis in children often occurs as a result of acute systemic inflammation, infection, and a high likelihood of central venous catheterization,^[20]^ whereas primary arterial thrombosis is relatively rare. Therefore, when encountering a cerebral embolism, the first thing that needs to be ruled out is a PFO-related paradoxical embolism.

Due to the severity and complexity of the condition of these 2 children, we finally performed occlusive sealing of the foramen ovale with the understanding and informed consent of the family. However, the fact is that there are no clear guideline recommendations for the treatment of children with PFO, and the specific choice of surgery or observation seems to be handled differently by different centers. New evidence emerges from recent clinical trials of unenclosed oval foramen to prevent secondary stroke.^[21]^ Among them, the guideline of the 2020 American Academy of Neurology Guidelines Subcommittee recommends that patients being considered for PFO closure who have had a stroke should confirm the location and size of the stroke lesion, refine angiographic imaging of the neck and intracranial area, refine the electrocardiogram for the presence of atrial fibrillation, and look for a higher mechanism contributing to the stroke, and perform a comprehensive evaluation to decide whether to perform closure of the PFO and state that after careful consideration of the risks and benefits of the procedure.^[22]^ It is reasonable to recommend closure of the PFO in patients under 60 years of age with embolic stroke who have no other obvious stroke mechanism. The European Society for Percutaneous Vascular Intervention considers that percutaneous PFO closure should be performed in patients between the ages of 18 and 65 years with a confirmed diagnosis of CS, transient ischemic attack, or systemic embolism.^[23]^ Using the same shared decision-making approach, PFO closure may also be considered in patients < 18 years of age, considering the lack of evidence, age-related confounders, and the additional risks of interventional and pharmacologic therapy. Therefore, we finally blocked the foramen ovale. However, whether it is effective in the long term, we will still be in the process of further follow-up. It has been noted that transcatheter PFO closure in children is a relatively safe and effective procedure, and long-term antithrombotic therapy is also a treatment option. However, long-term antithrombotic therapy is usually complex to maintain due to poor compliance and possible side effects.^[24]^ Some research indicates that cerebral ischemic events are rare in children. However, when they do occur, they can lead to permanent disability and require long-term medication to prevent recurrence.^[24,25]^ PE via PFO may be the cause of ischemic stroke. All pediatric and young adult patients with ischemic events should be evaluated for PE via PFO or atrial septal defect, and all patients should be extensively screened to exclude hypercoagulable states. Transcatheter device closure of PFOs and atrial septal defects is a safe alternative treatment option. In both cases, infarcts were identified on magnetic resonance imaging and diagnosed as PFO by ultrasound. After excluding carotid stenosis, myocarditis, hypercoagulability due to lupus, hemoglobinopathy, and other pathologies, PFO occlusion was performed when indicated.

In conclusion, we report 2 cases of unclosed foramen ovale with PE in children. If children are found to have unexplained cerebral infarctions, they should be routinely screened for PFO. Then, they should be evaluated accordingly and decide on the treatment plan. PFO occlusion or conservative treatment. Adult management programs are often used since no management program exists for children with PFO. We look forward to future extensive multicenter prospective studies to develop protocols for managing children with PFO.

## Author contributions

**Conceptualization:** Changjian Li.

**Data curation:** Jie Gong.

**Funding acquisition:** Jie Gong.

**Investigation:** Jie Gong.

**Methodology:** Jie Gong, Huayong Zhang.

**Resources:** Huayong Zhang.

**Supervision:** Yong Zhang.

**Validation:** Changjian Li.

**Writing – original draft:** Jie Gong.

**Writing – review & editing:** Yong Zhang.
